# The Missing Millions: A Threat to the Elimination of Leprosy

**DOI:** 10.1371/journal.pntd.0003658

**Published:** 2015-04-23

**Authors:** William Cairns Smith, Wim van Brakel, Tom Gillis, Paul Saunderson, Jan Hendrik Richardus

**Affiliations:** 1 School of Medicine and Dentistry, University of Aberdeen, Aberdeen, United Kingdom; 2 Netherlands Leprosy Relief, Amsterdam, Netherlands; 3 effect: hope, The Leprosy Mission, Markham, Ontario, Canada; 4 American Leprosy Missions, Greenville, South Carolina, United States of America; 5 Erasmus MC, University Medical Center, Rotterdam, Netherlands; London School of Hygiene and Tropical Medicine, UNITED KINGDOM

## Introduction

Leprosy is a slow, chronic disease with a long incubation period caused by *Mycobacterium leprae*. The clinical presentation varies across a wide spectrum from tuberculoid to lepromatous leprosy. The condition is characterized by skin lesions and damage to peripheral nerves leading to physical disability and social problems. The past 50–60 years have witnessed remarkable progress in the fight against leprosy. The introduction of dapsone therapy in the late 1940s was the first effective treatment for leprosy, and this was followed by the move to short course multidrug therapy (MDT) in 1981. The World Health Assembly Resolution in 1991 [1] to “eliminate leprosy as a public health problem” by the year 2000 galvanised extraordinary international support resulting in the fall in the point prevalence of patients registered for treatment of leprosy by over 90% to less than 1 in 10,000 at the global level. The effort was led by the World Health Organization (WHO) and supported by national governments and their health service staff, the Nippon Foundation, Novartis, the International Federation of Anti-Leprosy Organizations (ILEP), local non-governmental organizations (NGOs), and by people affected by leprosy. Since 2000, the focus has moved from prevalence of leprosy to incidence as measured by reported new case detection to sustain the achievements and to reduce the burden of disease, particularly on reduction and prevention of disability associated with leprosy and rehabilitation of those facing the long-term consequences of the disease [2].

## Understanding Transmission

Despite this remarkable progress, understanding of the pathogenesis of leprosy has remained unclear. Basic knowledge of the transmission of *M*. *leprae*, portals of exit and entry, the role of the environment and animal reservoirs, the development of immune responses following infection, and the pathogenesis of *M*. *leprae* infection to the disease of leprosy are all limited. A recent expert group, hosted by effect: hope (The Leprosy Mission Canada) and the National School of Tropical Medicine at Baylor College of Medicine in Houston, Texas, United States, reviewed the evidence and recent research on transmission and how to block it. Novel methods in strain typing *M*. *leprae* and recent findings in both host genetics and immune responses open the potential for new solutions. However, the very long incubation period, the very low incidence rates in those exposed, and the insidious clinical presentation create real challenges to developing strategies to interrupt transmission [3].

## Global Trends in Leprosy

**Fig 1:**
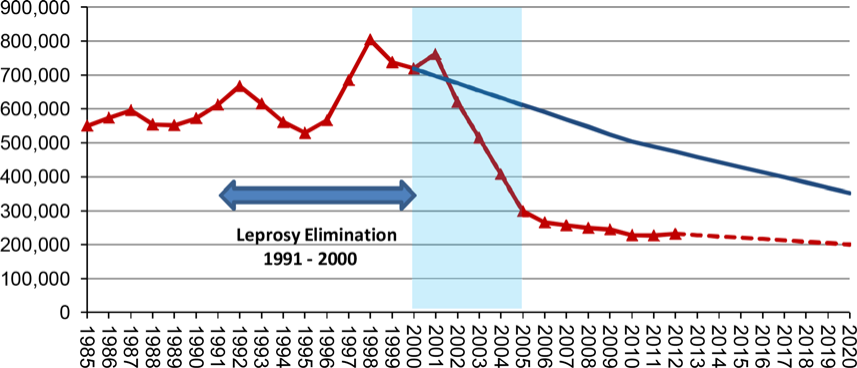
Number of new leprosy cases by year. The red continuous line represents the observed annual new case detection rate between 1985 and 2012, with extrapolation to 2020 based on the trend after 2005 (red dotted line). The blue continuous line is the predicted new case detection rate based on modeling with the SIMLEP model, applying an intermediate scenario in the presence of an infant BCG vaccination programme [7].

Global data on the trends in new case detection in leprosy are collated and published annually by WHO [4]. There are concerns about the quality and completeness of these data [5,6]. These data describe new case trends from detection through the completion of MDT at national, regional, and global levels. Fig 1 plots the number of new leprosy cases by year. The red continuous line represents the observed annual new case detection rate between 1985 and 2012, with extrapolation to 2020 based on the trend after 2005 (red dotted line). The blue continuous line is the predicted new case detection rate based on modeling with the SIMLEP model, applying an intermediate scenario in the presence of an infant BCG vaccination programme [7]. These trends in the last decade show a very striking feature (Fig 1, red line): a dramatic and sudden decline in new case detection of over 60% over a short period of time (2001–2005). Understanding the possible explanations for this dramatic fall is very important. One explanation is that this represents a true fall in the incidence of leprosy following reduction in transmission of *M*. *leprae* infection. Disease modeling work [7] has suggested that the long-term underlying trend in leprosy incidence in a good scenario including infant BCG immunization is a slow, gradual decline of around 4.4% per year. A large, sudden fall in transmission seems biologically implausible given the long and variable incubation period in leprosy and the evidence of continuing, significant rates of new cases in children [4]. A second explanation is that there was substantial overdiagnosis of leprosy prior to 2001, which has inflated the previous levels of new case detection. This may be a factor to explain the peak of new case detection between 1996 and 2001, a period of intensified case detection activities [8], such as Leprosy Elimination Campaigns (LEC) and Special Action Projects for the Elimination of Leprosy (SAPEL). However, the new case detection trends between 1985 and 1996 are remarkably stable and sustained overdiagnosis seems unlikely over this period. The third, and most probable, explanation is that the dramatic fall in new case detection is a result of a decline in leprosy activities following the declaration of elimination as a public health problem globally, and in individual countries. This decline includes reduced intensity and coverage of case detection activities, community awareness, and training in the diagnosis and treatment of leprosy often associated with the move from vertical leprosy control activities to integrated approaches. The recent rise in disability in new cases detected and the increasing delay in diagnosis reported by many countries supports this explanation [4]. WHO, along with the Nippon Foundation, called an International Leprosy Summit in 2013 to address what they called “stagnation” in the leprosy control. This resulted in the Bangkok Declaration [9], signed by the health ministers of the major leprosy endemic countries, calling for renewed political commitment to leprosy control.

## The Implications of the Decline in New Case Detection for the Elimination of Leprosy

Fig 1 shows how the model prediction of the long-term trend in new leprosy case detection based on the observed figures before 2000 compares with the observed new case detection. The resulting difference between the expected and observed numbers of new cases of leprosy between 2000 and 2012 is approximately over 2.6 million. This number will increase to over 4 million by 2020. This analysis implies that there may be a large accumulation of people with leprosy in the community who remain undiagnosed and untreated. This assertion is supported by evidence from recent sample surveys in endemic areas detecting many as yet untreated cases in Bangladesh [10] and in India [11]. This large number of undetected cases represents a major threat to leprosy control and contributes to the increased burden of infection in the community and an increased pressure on transmission. This has major consequences for the road map for NTDs in the London Declaration [12–14], which targets “interruption of transmission” and “global elimination” of leprosy by 2020.

## Response to This Threat to Leprosy Elimination

It is vital that all involved and concerned with leprosy control appreciate this situation and recognise that the London Declaration targets of “global elimination” of leprosy and “interruption of transmission” by 2020 are likely to be unobtainable and that revised targets are needed. Major commitments and resources need to be made available without delay. While local elimination (based on new cases detected in a defined locality) of leprosy through targeted leprosy control activities as recommended by WHO is necessary in the short-term, the complete interruption of transmission at a global level will require new tools based on game-changing discoveries. A significant investment in complementary research efforts, designed to better understand the basic elements of transmission, is necessary for achieving “interruption of transmission.”

The development of collaboration with other NTD programmes represents a real opportunity to improve the coverage, quality, and cost-effectiveness of leprosy control with numerous cross-cutting opportunities in drug delivery, surveillance, training, disability prevention, and morbidity management. The commitment called for by health ministers in the Bangkok Declaration is also essential at all levels, internationally, nationally, and locally by national governments and by all agencies that support national programmes, including Governmental and non-governmental agencies, industry, and people affected by leprosy. The global introduction of post-exposure prophylaxis [15–17] is a real opportunity to re-energise leprosy control activities through increased community awareness, capacity building, and active management of contacts. The research opportunity recently launched by the Leprosy Research Initiative leads the way to develop further innovations for leprosy control, but much more support is needed for basic, as well as operational, research to develop strategies to interrupt transmission. For example, recent findings have revealed new insights into zoonotic relationships, genetic markers for host susceptibility and resistance, as well as environmental factors that continue to test our long-held notions of the ecology of *M*. *leprae* and leprosy. Understanding these relationships may provide the knowledge to move from management practices to strategies designed to stop transmission.

The WHO priority to promote early detection and to monitor this through measuring disability in new case detection is a vital component to evaluate enhanced initiatives designed to reduce transmission. However, addressing the gap between the incidence and case detection of leprosy requires improved strategies for case detection, new tools for early diagnosis, and major efforts to improve community awareness and capacity of health staff to diagnose and manage leprosy and its complications.

The challenge is to tackle the research gaps through novel collaborations, to improve operational collaborations with multiple players in all NTDs, and to incorporate new approaches in community engagement that would enhance public health at the community level. The leprosy world, including WHO, national governments, NGOs, the research community, and industry, together with people affected by leprosy, must respond to this situation that, if left unaddressed, could see all the past achievements in leprosy control reversed.
